# Clinical and molecular characterization of hepatic glycogen storage disease in Saudi Arabia

**DOI:** 10.1371/journal.pone.0329008

**Published:** 2025-07-31

**Authors:** Abdulrahman Al-Hussaini, Mohammed AlMannai, Muhannad Alruwaithi, Eissa Faqeih, Ali Alasmari, Majid Alfadhel, Fuad Al Mutairi, Mohammed Saleh, Abdullah AlZaben, Yaser Alobailan, Moodhi Alharbi, Manal AlAfqi, Alaa Alayed, Abdul Ali Peer-Zada, Yasir Alrusayni

**Affiliations:** 1 Division of Pediatric Gastroenterology, Children’s Specialized Hospital, King Fahad Medical, City Riyadh, Saudi Arabia; 2 College of Medicine, Alfaisal University, Riyadh, Saudi Arabia; 3 Prince Abdullah Bin Khaled Celiac Disease Research Chair, Department of Pediatrics, Faculty of Medicine, King Saud University, Riyadh, Saudi Arabia; 4 Medical Genomic Research Department, King Abdullah International Medical Research Center (KAIMRC), King Saud Bin Abdulaziz University for Health Sciences (KSAU-HS), Riyadh, Saudi Arabia; 5 Genetics and Precision Medicine department (GPM), King Abdullah Specialized Children’s Hospital (KASCH), King Abdulaziz Medical City, Ministry of National Guard Health Affairs (MNG-HA), Riyadh, Saudi Arabia; 6 Division of Medical Genetics, Specialized Children’s Hospital, King Fahad Medical City, Riyadh, Saudi Arabia; 7 Division of Pediatric Gastroenterology, Department of Pediatrics King Abdullah Specialized Children’s Hospital, King Abdulaziz Medical City, Ministry of National Guard Health Affairs (MNG-HA), Riyadh, Saudi Arabia; 8 Division of pediatric gastroenterology, Maternity & Children’s hospital, Buraydah, AlQassim, Saudi Arabia; 9 Division of Medical Genetics King Salman Medical City, AlMadinah AlMunawarah, Saudi Arabia; 10 Genetic Department, Maternity & Children’s Hospital, Buraydah, AlQassim, Saudi Arabia; 11 Molecular Pathology section, Pathology and Clinical Laboratory Medicine Administration King Fahad Medical City, Riyadh, Saudi Arabia; 12 Pathology section, Pathology and Clinical Laboratory Medicine Administration King Fahad Medical City, Riyadh, Saudi Arabia; Sohag University Faculty of Medicine, EGYPT

## Abstract

**Background and objectives:**

The paucity of data on glycogen storage diseases (GSDs) from Arabs prompted us to report on hepatic GSD to characterize its clinical and molecular features and outcomes among Saudi children and to evaluate genotype‒phenotype correlations.

**Methods:**

We retrospectively reviewed the charts of 65 children (37 females) with genetically confirmed hepatic GSD who presented between 2008 and 2020 and were followed up for a median duration of 9 years (range: 0.4–21 years).

**Results:**

The most common hepatic GSD in our cohort was GSD Ia (37%), followed by GSD III (20%), GSD Ib (12.3%), and GSDVI (10.8%). Twenty-seven variants were identified (8 novel and 4 from the common ancestor, i.e., “founder in nature”). The most common founder variant is P.(Arg83Cys) in the *G6PC1* gene (20% of the 65 GSD patients), clustering in Aseer Province. Six patients underwent liver transplantation (due to difficulty controlling hypoglycemia in 5 GSD Ia patients and severe portal hypertension in one GSD IV patient). One patient with GSD type 1b developed hepatic adenoma at the age of 17 years. A patient with GSD IXc developed portal hypertension at the age of 5 years, and one patient with GSD IXa developed cirrhosis. Renal complications developed in 18 patients. An echocardiogram was performed in 16 patients and revealed mild–moderate asymptomatic left ventricular hypertrophy in 5 patients. The majority of the hepatic GSD cases in our cohort manifested a severe phenotype (hepatomegaly, hypoglycemia, ± systemic involvement); only the 7 GSD VI patients manifested a mild phenotype (hepatomegaly without hypoglycemia). No “genotype‒phenotype correlations” could be observed when the two common *G6PC1* gene variants [p.(Arg83Cys) *versus* p.(Gln20Arg)] were compared.

**Conclusion:**

With the exception of GSD VI, all the hepatic GSD subtypes in Saudi Arabia are associated with a severe phenotype. Identification of the predominant gene variants and their geographic distribution in any population is likely to facilitate rapid molecular analysis by future targeting of that specific mutation.

## Introduction

Glycogen storage diseases (GSDs) constitute a group of rare inherited inborn errors in glycogen metabolism caused by deficiencies in enzymes or transporters, which are essential for the synthesis and degradation of glycogen [1]. There are various clinical phenotypes of GSD that are classified on the basis of the deficient enzymes and affected tissues into the following: hepatic form, muscular form (skeletal and/or cardiac muscle), or both. Hepatic GSD includes several genetically heterogeneous disorders (GSD 0, GSD I, GSD III, GSD IV, GSD VI, GSD IX, and GSD XI) with overlapping clinical, biochemical, and histological features, which makes the identification of a specific hepatic subtype of GSD challenging for clinicians. Therefore, the definite diagnosis of a hepatic subtype of GSD requires molecular genetic analysis or the demonstration of specific enzyme deficiency in liver tissue. It is important to identify the exact diagnosis to provide personalized care and appropriate family counseling, as these subtypes of hepatic GSD vary in their treatment, natural course, and outcome.

Over the past two decades, more knowledge has been gained on the pathophysiology, disease course, treatment, and complications associated with GSDs. These data originate from different populations and ethnicities [2–9]. Data on GSD among Arabs are scarce and limited to two reports on GSD III from Egypt and Tunisia [10,11]. The current knowledge on GSD in Saudi Arabia derives mainly from case reports on a single *PHKG2* mutation causing GSD IX [12] or small single-center case series on a single *AGL* mutation causing GSD III [13]. However, no reports have yet been published on other types of GSDs; particularly, data on long-term outcomes are lacking. The scarcity of data from Arabs prompted us to report 65 confirmed cases of various subtypes of hepatic GSD to characterize the epidemiological, clinical, laboratory, and molecular features and outcomes of these diseases among Saudi children and to evaluate genotype‒phenotype correlations.

## Methods and patients

### Study settings and design

This retrospective study was based on an analysis of data collected from patients’ electronic medical records. The study included 2 tertiary care centers in Riyadh city that receive patients with suspected GSD from different regions across Saudi Arabia.

### Study population

We identified all patients with confirmed hepatic GSD who were referred to either hospital between 2008 and 2020. The inclusion criteria were as follows: 1) under 14 years of age at the time of diagnosis and 2) confirmed diagnosis of hepatic GSD on the basis of genetic testing. The exclusion criterion included suspected GSD that was not confirmed via molecular testing.

### Study procedures

a) The medical records of the patients were accessed between 1/6/2020 and 31/12/2021 to collect the following data: (1) demographic and clinical characteristics; (2) laboratory data at presentation: total and direct bilirubin, alanine transaminase (ALT) [normal < 55 units/l], aspartate transaminase (AST) [normal < 34 units/l], gamma-glutamyl transferase [normal < 60 units/l], serum triglycerides (normal <1.7 mmol/L), serum cholesterol (normal <4.4 mmol/L), serum uric acid (normal <420 µmol/L), serum lactate (normal < 2.2 mmol/l), and creatine phosphokinase (normal <250 u/l); (3) imaging findings, including echocardiogram; (4) histopathological findings; (5) results of gene testing; (6) treatment provided; (7) complications that developed during the disease course; (8) date at last follow-up; and (9) final outcome. These data were compiled into an Excel spreadsheet.
b) Molecular genetic Investigations

Throughout the study period, blood samples were examined by single-gene sequencing, targeted analysis of pathogenic variants, next-generation sequencing (GSD panel), or whole-exome sequencing (WES). The identified variants were classified and interpreted according to the ACMG guidelines [14]. Briefly, ACMG criteria that applied to the genetic variants in the current study included PVS1 (pathogenic very strong), PM2 (pathogenic moderate), PM3 (pathogenic very strong), PM4 (pathogenic moderate), PM5 (pathogenic moderate), PP2 (pathogenic supporting), PP3 (pathogenic moderate), PP5 (pathogenic supporting), and BP6 (benign supporting). All the variants were checked in the KFMC-in-house allele frequency (MAF) database (4500 WES cases containing 2.2 million variants) to rule out polymorphisms (MAF > 1%). For variants of unknown clinical significance (VUS) and novel variants, conservation across species was assessed as additional evidence for pathogenicity. Where available, parental blood DNA samples were analyzed for familial segregation by Sanger sequencing. All procedures were conducted with informed consent.

c) Histopathological studies

The liver samples were fixed in 10% buffered formaldehyde, paraffin-embedded, and stained with hematoxylin and eosin, Masson’s trichrome stain for fibrous tissue and Perls’ method for iron, reticulin, and periodic acid–Schiff diastase. The biopsy was evaluated for the presence of distension of hepatocytes with glycogen, steatosis, glycogenated nuclei, and portal fibrosis (stage 0: no fibrosis; stages I–II: mild fibrosis; stages III–IV: advanced fibrosis).

#### Study outcomes.

- The primary outcomes included the following: 1) survival with native liver (defined as starting at birth and ending at death, liver transplantation (LT), or last follow-up in patients with native liver), 2) need for LT, and 3) death due to complications of the disease.
- The secondary outcomes included a) the development of complications during the disease course and b) the presence of genotype‒phenotype correlations.

### Clinical phenotype

The clinical phenotype of the disease was categorized into the following groups on the basis of the clinical presentation, disease course, and outcome at the last follow-up:

a) “Mild phenotype” (defined as the presence of hepatomegaly without hypoglycemia, systemic complications, or progression to advanced liver disease)
b) “Severe phenotype”, if any one of the following occurred:
  - Hepatomegaly and difficulty in controlling hypoglycemic episodes that require continuous overnight feeding, cornstarch during sleeping, or LT.
  - Progression of liver disease to cirrhosis, as evident by Grade III/IV fibrosis on liver biopsy; development of portal hypertension, as evident by splenomegaly and thrombocytopenia [platelet count < 150,000/ml]; or development of upper gastrointestinal bleeding due to esophageal or gastric varices)
  - Hepatomegaly with mild hypoglycemic episodes and the development of a systemic complication (kidney, heart, or bone disease, neurodevelopmental delay, short stature).
c) “Intermediate phenotype”, a status between mild and severe phenotypes characterized by hepatomegaly and mild hypoglycemia without the development of systemic complications or advanced liver disease.

### Ethical consideration

The local review board of King Fahad Medical City approved the study (IRB Log number 17–093). Owing to the retrospective design of the study, the ethics committee waived the requirement for informed consent.

### Statistical analysis

The data analyses were performed using the Statistical Package for Social Sciences, version 26 (SPSS, Armonk, NY: IBM Corp.). Descriptive statistics are presented as numbers, percentages, means and standard deviations. The relationships between the type of GSD and the different characteristics of the patients were determined using Fisher’s exact test for dichotomous data. Differences between continuous data were analyzed using the unpaired two-tailed t test for normally distributed continuous data and the Mann‒Whitney U test or Kruskal‒Wallis test for nonnormally distributed data. A P value <0.05 was considered to indicate statistical significance.

## Results

### General characteristics of the study cohort

**Fig 1:**
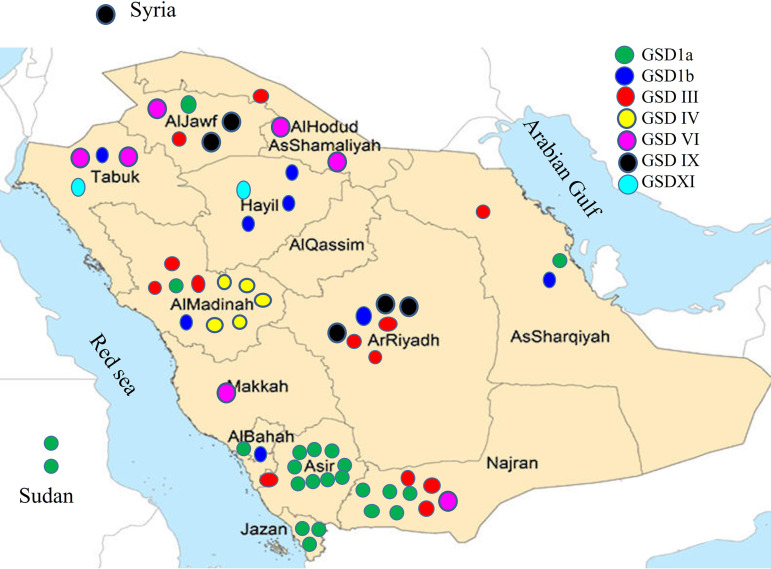
Geographic distribution of the 65 cases of hepatic GSD in Saudi Arabia. The figure is similar but not identical to the original image and is therefore for illustrative purposes only.

During the study period, 81 patients were evaluated for the possibility of hepatic GSD. The diagnosis of hepatic GSD was confirmed in 65 patients (61 families; 62 were Saudi, two were Sudanese and one was Syrian; 37 females). The median age at first presentation was 10 months (range: 1 month–12 years). A diagnosis was made during the neonatal period in 3 patients [GSD 1(a), GSDIII, GSD IV)] on the basis of screening of an affected older sibling. A family history of GSD was positive in 28% of the 62 families, and consanguinity was observed in 75%. The most common type of hepatic GSD in our cohort was GSD Ia (37%), followed by GSD III (20%), GSD Ib (12.3%), and GSDVI (10.8%). GSD types IX (9.2%), IV (7.7%) and XI (3%) were less common in our cohort. Overall, the most common presenting clinical finding was hepatomegaly (84.5%), followed by hypoglycemia (39.7%). The median age at the time of the last follow-up was 9 years (0.4–21 years). Fig 1 shows the geographic distribution of hepatic GSD in the 13 provinces of Saudi Arabia. One-third of the cases (31%) originated from the southwestern region with prominent clustering of GSD1a, and 25% of the cases originated from the northern provinces without obvious clustering of a specific type. All 5 GSD IV cases were from 5 unrelated families from one tribe in Al Madinah AlMunawarah Province.

### Clinical Phenotype

Tables 1,2 show a comparison of several clinical and laboratory variables related to hepatic GSD in our cohort. An earlier age of onset was more common in GSD Ia and GSD 1b patients, whereas a later age of onset was more common in GSD III and GSD VI patients (**P* *< 0.001). The mean values of lactate (8.22 ± 4.84, *p* = 0.001), uric acid (386.1 ± 141.4, *p* = 0.031) and serum triglyceride (8.90 ± 9.28 *p* = 0.001) were significantly greater in GSD ty*p*e Ia patients, whereas the mean values of ALT (525.9 ± 301.3, p < 0.001), AST (721.8 ± 788.4, *p* = 0.008) were significantly greater in GSD ty*p*e III patients. The median serum CPK level was significantly greater in the GSD III group than in the GSD Ia, GSD Ib, and GSD VI groups [557 U/L, (60–2086), *p* < 0.001]. No statistically significant differences were observed in the com*p*arisons of other variables. Types IV, IX, and XI were less common in our cohort (Table 2). The ALT value at presentation was normal in 16 of the 65 patients (25%) [GSD Ia = 6, GSD Ib = 4, GSD VI = 3, GSD IX = 1, GSD XI = 2]; 4 of the 16 patients presented elevated ALT levels at follow-up. Ten of the 49 patients with elevated ALT at presentation (21%) had normal ALT at the time of last follow-up.

**Table 1:** Clinical presentation of the most common hepatic GSDs.

Variables	GSD Ia(n = 24) (%)	GSD Ib (n = 8) (%)	GSD III (n = 13) (%)	GSD VI (n = 7) (%)	P-value^§^
**Age group in months at presentation**					
≤15 months	24 (100%)	7 (87.5%)	7 (53.8%)	1 (14%)	<0.001**
>15 months	0	1 (12.5%)	6 (46.2%)	6 (86%)	
**Clinical findings at presentation**					
Progressive abdominal distention	5 (20%)	3 (37.5%)	4 (30.8%)	7 (100%)	
Hepatomegaly	24 (100%)	8 (100%)	12 (92.3%)	7 (100%)	0.90
Hypoglycemia	16 (66.7%)	3 (37.5%)	6 (46.2%)	0	0.007**
Delayed motor milestone	3 (12.5%)	2 (25%)	0	0	0.271
Short stature	3 (12.5%)	1 (12.5%)	2 (15.4%)	0	1.000
Recurrent attacks of convulsion	4 (16.7%)	1 (12.5%)	2 (15.4%)	0	0.935
**Mean serum values at presentation**					
ALT (I/U) (normal <55)	138.0 ± 154.4	58.8 ± 32.5	525.9 ± 301.3	134.4 ± 121.1	<0.001**
AST (I/U) (normal <34)	295.8 ± 680.7	82.6 ± 73.9	721.8 ± 788.4	147.8 ± 119.9	0.111
ALP (I/U) (normal 140–460)	300.3 ± 175.8	248.4 ± 75.5	321.6 ± 80.5	325.4 ± 62.1	0.536
GGT (I/U) (normal 10–100)	173.1 ± 117.1	71.8 ± 71.4	270.3 ± 501.3	48.7 ± 12.7	0.603
Total bilirubin (µmol/L) (normal 1.7 to 20)	8.68 ± 11.9	3.21 ± 1.26	10.3 ± 4.02	15.9 ± 16.0	0.118
Direct bilirubin (µmol/L) (normal 0–8)	5.90 ± 8.80	1.33 ± 5.69	2.70 ± 0.96	6.88 ± 6.53	0.478
Triglycerides (mmol/L) (normal <1.7)	8.90 ± 9.28	4.23 ± 2.59	4.19 ± 1.99	3.31 ± 1.11	<0.001**
Cholesterol (mmol/L) (normal <4.4)	4.39 ± 1.99	3.63 ± 1.61	5.63 ± 1.75	4.84 ± 1.29	0.103
Creatine phosphokinase (U/L) (normal <170)	75 (36 - 103	71 (54–89)	557 (60 - 2086)	74 (21.2 - 125	< 0.001**
Uric acid (µmol/L) (normal 210–420)	386.1 ± 141.4	247.6 ± 132.6	244.6 ± 54.7	224.3 ± 28.0	0.003**
Lactate (mmol/L) (normal 0.5–2.2)	8.22 ± 4.84	4.27 ± 1.74	2.13 ± 2.02	2.50 ± 1.87	<0.001**

^§^P-value has been calculated using Fischer Exact test.

** Significant at p < 0.05 level.

**Table 2:** Clinical and laboratory features of the hepatic GSD IV, GSD IX, and XI.

Variables	GSD IV N = 5	GSD IX N = 6	GSD XI N = 2
**Median age at presentation (mo)**	20	17.5	6
**Clinical findings at presentation**			
Hepatomegaly	5 (100%)	6 (100%)	2 (100%)
Hypoglycemia	3 (60%)	4 (66.7%)	1 (50%)
Delayed motor milestone	1 (20%)	2 (33%)	2 (100%)
Short stature	0	2 (33%)	2 (100%)
Recurrent attacks of convulsion	1 (20%)	1 (12.5%)	0
**Median serum values at presentation**			
ALT (I/U) (normal <55)	171 (67–429)	165 (50–752)	37 (34-40)
AST (I/U) (normal <34)	200 (47–644)	222 (66-875)	51 (36-66)
ALP (I/U) (normal 140–460)	268 (236–350)	353 (239-1123)	977 (959-995)
GGT (I/U) (normal 10–100)	71 (16-113)	115 (7-161)	–
Total bilirubin (µmol/L) (normal 1.7 to 20)	5.8 (3.2-7.1)	9.6 (4-22.6)	–
Direct bilirubin (µmol/L) (normal 0–8)	4.1 (1.8-6.5)	–	–
Triglycerides (mmol/L) (normal <1.7)	1.29 (0.7–2.3)	1.79 (1.53-4.9)	1.9 (0.63-3.2)
Cholesterol (mmol/L) (normal <4.4)	3 (2.7-3.4)	4.94 (4.1-6.7)	4.55 (3.2-4.8)
Creatine phosphokinase (U/L) (normal <170)	123 (36-150)	21 (19-105)	–
Uric acid (µmol/L) (normal 210–420)	203 (1.8-267)	155 (93-229)	50
Lactate (mmol/L) (normal 0.5–2.2)	1.7 (0.9-2.5)	1.3	2.35 (1.6-3.1)

### Systemic involvement/complications

Table 3 summarizes the complications/comorbidities associated with the GSD patients in our cohort that developed over the follow-up period. A total of 6 patients (9.2%) underwent LT. Five patients had GSD1a [four harboring c.247C > T (p.Arg83Cys), one harboring c.620del (p.Lys207Argfs*26)] associated with poor metabolic control, severe hypoglycemia and growth failure (median age of 5 years; range: 0.5–5 years), and one patient with GSD IV was transplanted at the age of 2 years because of worsening portal hypertension. One patient with GSD type 1b developed hepatic adenoma at the age of 17 years (S1 Fig) and needed surgical resection. Another patient with GSD IX [*PHKG2*: c.913dup (p.Arg305Profs*84)] developed portal hypertension at the age of 5 years. Gallbladder stones were observed in one of the 7 patients with GSD VI.

**Table 3:** Systemic involvement and complications associated with GSD-subtypes over the follow up period.

Type of GDS	Systemic involvement	Median age at last follow up (year)
**GSD Ia** (n = 24)	One case mild dilated left ventricle with mild aortic valve insufficiency 54% enlarged echogenic kidneys 8% chronic kidney disease One case nephrocalcinosis 8% Nephrolithiasis 17% short stature 12.5% neurodevelopmental delay **21%** Liver transplant	9
**GSD Ib (n** = 8)	63% Neutropenia ± chronic diarrhea and recurrent infections 12.5% Hepatic adenoma (at age 17 years) 12.5% nephrolithiasis 12.5% chronic kidney disease 25% neurodevelopmental delay with intellectual disability 65% short stature	10
**GSD III** (n = 13)	15% mild-moderate left ventricular hypertrophy 1/13 Bone dysplasia 28% short stature 14% neurodevelopmental delay	8
**GSD IV** (n = 5)	20% dilated left ventricle and left atrium with mild-moderate mitral regurgitation 40% short stature 20% neurodevelopmental delay Portal HTN in one case and needed LTx at age 2	6
**GSD VI** (n = 7)	One case mild left ventricular hypertrophy One case Celiac disease One case asymptomatic gallbladder stone	10
**GSD IX** (n = 6)	33% Neurodevelopmental delay with intellectual disability 33% Short stature Hydronephrosis Portal HTN (at age 5 years)	12
**GSD XI** (n = 2)	Both cases had renal tubular acidosis and rickets One is short One with Echogenic kidneys	6

Renal complications developed in 18 of the 65 hepatic GDS patients (28%): 13 GSD 1(a) patients developed large echogenic kidneys, including one patient with nephrocalcinosis and two with nephrolithiasis, and two patients developed chronic kidney disease resulting in renal failure. Renal involvement in the 2 patients with GSD XI was in the form of proximal renal tubular acidosis. An echocardiogram was performed in 16 patients and revealed abnormalities in 5 patients (31%) in the form of asymptomatic mild–moderate left ventricular hypertrophy not requiring medical therapy. Neurodevelopmental delay with intellectual disability was a feature in some patients with GSDs I, III, IV, or IX. Neutropenia was a unique common feature (65%) in GSD I(b) patients, leading to recurrent infections. Growth impairment was a common manifestation in GSD I(b) and occurred less frequently in other forms of hepatic GSD.

### Liver histopathology

Liver biopsy was performed in 13 patients (median age at biopsy: 2.5 years, range: 8 months–8 years) (S1 Table). All the biopsies revealed hepatocyte distension with glycogen and variable degrees of glycogenated nuclei and steatosis. The stage of portal fibrosis was 0–1 in the GSD I (S2 Fig) and GSD VI patients (S3 Fig) who underwent liver biopsy. The most common findings were distension of hepatocytes with glycogen (93.75%), glycogenated nuclei (54%), fatty infiltration (38.5%), and portal fibrosis I/II (56.25%). Two patients with GSD III underwent liver biopsy, and the stage of fibrosis ranged between 3 and 4. Portal fibrosis ranged from stage 2–4 in the 3 patients with GSD IX (stage 2 in one patient with *PHKB* and stage 3–4 in *PHKA2* [S4 Fig] and *PHKG2*, one each).

### Molecular analysis

**Fig 2:**
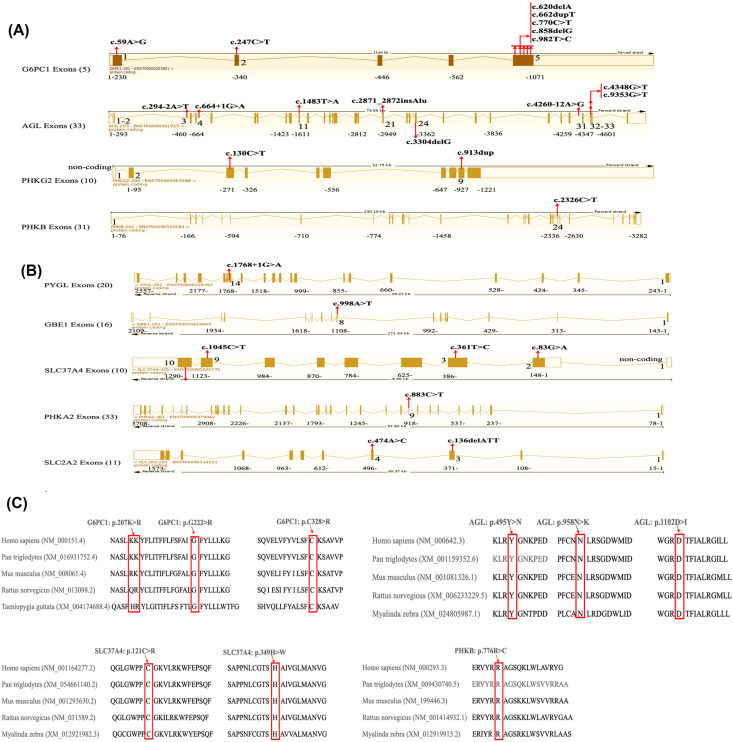
Exon map of gene variants identified in GSD patients (A, forward strand; B, reverse strand) and conservation of mutated amino acids across species (C, novel variants and VUS only). (A) Detailed exon map of genes in the forward direction, with the solid bars representing exons (left to right) and arrows showing the variants at specific exons. The number beside the gene name represents the total number of exons in that particular gene. (B) Detailed exon map of genes in the reverse direction, with the solid bars representing exons (right to left) and arrows showing the variants at specific exons. The number beside the gene name represents the total number of exons in that particular gene. (C) Only VUS and novel variants are shown here. Part of the gene nucleotide sequence obtained from NCBI showing the conservation of the mutated amino acid (see arrows) across species.

Fig 2 presents the exon map of the 27 homozygous mutations identified in the 9 genes causing hepatic GSD (8 are novel variants). S5 Fig shows the distribution of the GSD subtypes and variants among the 65 Saudi children. For further confirmation of the pathogenicity of the variants, parental testing was performed using targeted mutational analysis. The most common mutation type was missense (13 variants = 48%), followed by small deletions or duplications resulting in frameshifts (6 variants = 22%), splice site mutations (4 variants = 15%) predicted to be splice acceptor site mutations, nonsense mutations resulting in premature stop codons (3 variants = 11%), and only one in-frame deletion and insertion of an Alu repeat (Table 4). The most commonly encountered variants were c.247C > T p.(Arg83Cys) and c.59A > G, p.(Gln20Arg) in the *G6PC1* gene, c.1768 + 1 G > A in the *PYGL* gene, and c.998A > T, p.(Glu333Val) in the *GBE1* gene. These variants occurred in apparently unrelated families from several tribes in Saudi Arabia, indicating that these mutations probably came from common origins, i.e., “founder gene mutations”. The two founder *G6PC1* variants originated from the southwestern region of Saudi Arabia; specifically, the variant p.(Arg83Cys) was the predominant variant in Aseer Province, and the variant p.(Gln20Arg) was the only variant in the GSD Ia cases from Najran Province.

**Table 4:** Hepatic GSD genetic variants (n = 65 patients, 61 families, 27 variants).

Gene	No. Family (no. of patients)	Variant	Type of Variant	ACMG classification (criteria)	Clinical phenotype
***G6PC1***	12 (13)	**1. NM_000151.4:c.247C > T, p.(Arg83Cys)**	Missense	Pathogenic (PM1, PM2, PM3, PM5, PP2, PP3)	Severe
	4 (5)	**2. NM_000151.2:c.59A > G, p.(Gln20Arg)**	Missense	Likely pathogenic, (PM2, PM3, PP2, PP3)	Severe
	1 (2)	3. NM_000151.4:c.770C > T, p.(Pro257Leu)	Missense	Likely pathogenic, (PM1, PM2, PM5, PP2, PP3)	Mild
	1 (1)	4. NM_000151.4:c.858delG, p.(Lys287AsnfsTer14)	Frameshift	Likely Pathogenic. (PVS1, PM2, PP5)	Severe
	1 (1)	5. NM_000151.3:c.662dupT, p.(Gly222ArgfsTer21)⁑	Frameshift	Likely Pathogenic, (PVS1, PM2, PP5)	Severe
	1 (1)	6. NM_000151.2:c.620delA, p.(Lys207ArgfsTer26)⁑	Frameshift	Likely pathogenic, (PVS1, PM2)	Severe
	1 (1)	7. NM_000151.4: c.982T > C; p.C328R ⁑	Missense	Likely Pathogenic, (PM1, PM2, PP2, PP3)	Severe
***SLC37A4***	3 (3)	1. NM_001164277.1:c.83G > A, p.(Arg28His)	Missense	Likely Pathogenic, (PM1, PM2, PM5, PP2, PP5)	Severe
	3 (3 cousins)	2. NM_001164277.1:c.1243C > T p.(Arg415Ter)	Nonsense	Pathogenic (PVS1, PM2, PM3)	Severe
	1 (1)	3. NM_001164278.1:c.1045C > T, p.(His349Tyr)	Missense	VUS (PM2, PP2, BP6)	Severe
	1 (1)	4. NM_001164277.1:c.361T > C, p.(Cys121Arg)⁑	Missense	VUS (PM1, PM2,PP2)	Severe
***AGL***	3 (3)	1. NM_000082.2:c.4353G > T, p.(Trp1451Cys)	Missense	Pathogenic (PM2, PM3, PP3, PP5)	Intermediate
	3 (3)	2. NM_000642.2:c.4260-12A > G	Splice site	Likely Pathogenic (PM2, PM3, PP5)	Severe
	2 (2 cousins)	3. NM_000642.2:c.664 + 1G > A	Splice site	Likely Pathogenic (PVS1, PM2, PP5)	Severe
	1 (1)	4. NM_000642.2:c.3304delG, p.(Asp1102IlefsTer11)⁑	Frameshift	Likely Pathogenic, (PVS1, PM2)	Severe
	1 (1)	5. NM_000028.2:c.294-2A > T	Splice site	Pathogenic (PVS1, PM2, PM3, PP5)	Severe
	1 (1)	6. NM_000642.2:c.4348G > T, p.(Glu1450Ter)	Nonsense	Pathogenic (PVS1, PM2, PM3, PP5)	Severe
	1 (1)	7. NM_000642.2:c.1483T > A, p.(Tyr495Asn)⁑	Missense	VUS (PM2, PP3)	Mild
	1 (1)	8. NM_000642.2:c.2871–2872insAlu (p.Asn958Lysfs*2)⁑	Insertion of Alu repeat	Likely pathogenic, (PVS1, PM2)	Severe
***GBE1***	5 (5)	**1. NM_000158.4:c.998A > T, p.(Glu333Val)**	Missense	Pathogenic (PM2, PM3, PP3, PP5)	Severe
***PYGL***	7 (7)	**1. NM_002863.4:c.1768 + 1 G > A**	Splice site	Pathogenic (PVS1, PM2, PM3, PP5)	Mild
***PHKG2***	2 (3)	1. NM_000294.2:c.130C > T, p.(Arg44Ter)	Nonsense	Pathogenic (PVS1, PM2, PP5)	Severe
	1 (1)	2. NM_000294.2:c.913dup, p.(Arg305Profs*84)	Frameshift	Likely Pathogenic (PVS1, PM2)	Severe
***PHKA2***	1 (1)	1.NM_000292.2:c.883C > T, p.(Arg295Cys)	Missense	Pathogenic (PM2, PM3, PM5, PP3, PP5)	Severe
***PHKB***	1 (1)	1.NM_001031835.3:c.2326C > T, P.(Arg776Cys)⁑	Nonsense	VUS (PM2, PP3, BP6)	Intermediate
***SLC2A2***	1 (1)	1. NM_001278658.1:c.134_136delATT, p.(Tyr45del)	In-frame deletion	VUS (PM2, PM4)	Severe
	1 (1)	2. NM_000340.1:c.474A > C, p.(Arg158Ser)	Missense	VUS (PM2, PP3, PP5)	Severe

Bolded variants are the founder gene mutations; ⁑ indicates the novel variants

The vast majority of the hepatic GSD cases in our cohort manifested a severe phenotype, with few exceptions. All 7 unrelated GSD VI patients (harboring the founder *PYGL* variant c.1768 + 1 G > A from different regions in Saudi Arabia) manifested a mild phenotype characterized by hepatomegaly without hypoglycemia or significant complications/comorbidities (Table 4). Among the 7 variants causing GSD I(a), a missense variant [c.770C > T, p.(Pro257Leu)] led to a mild phenotype. Among the 8 variants causing GSD III, two missense variants [p.(Tyr495Asn) in one patient and p.(Trp1451Cys) in 3 unrelated patients] led to a mild (hepatomegaly and elevated liver enzymes alone without hypoglycemia over a 5-year follow-up period) and intermediate phenotype (hepatomegaly and mild hypoglycemia without the development of systemic complications or advanced liver disease over a follow-up period of 14–16 years), respectively (Table 4). Further subanalysis of patients with GSD Ia did not reveal a clear genotype‒phenotype correlation (S2 Table). Five of the GSD IX patients (*PHKG2* = 4, *PHKA2 *= 1) manifested a severe phenotype, and one patient with a *PHKB* variant manifested an intermediate phenotype over a median 12-year follow-up period.

### Treatment

The most common treatment modality used was uncooked cornstarch (76.5% of all the subjects, 94.4% of the subjects with GSD I), followed by frequent carbohydrate meals (67.6%). A high-protein diet and restriction of lactose and fructose were recommended for all patients with hypoglycemia. Allopurinol was used for hyperuricemia in 55.6% of the children with GSD Ia. Sodium or potassium acetate was used to treat the accompanying acidosis. Neutropenia was documented in 5 of the 8 patients with GSD Ib, and G-CSF was used for severe neutropenia in four patients.

### Long-term survival of patients with hepatic GSD

In addition to the 6 patients (9.2%) who underwent LT, only patients with GSD III (1.5%) died at the age of 2 years due to sepsis. The remaining 58 patients (89%) were still alive with a native liver [median age at the time of the last follow-up was 9 years (range: 0.4–21 years)].

## Discussion

Our study is the first study from the Arabian Peninsula that presents a comprehensive analysis of hepatic GSD in a large sample of Saudi patients. Our study highlights important observations. First, our molecular analysis confirmed that hepatic GSDs (particularly GSD Ia, GSD Ib, GSD III, GSD IX and GSD XI) are highly genetically heterogeneous disorders with a large spectrum of mutations in the same population. On the other hand, the 5 unrelated families with GSD IV and the 7 unrelated families with GSD VI harbored a single pathologic variant, p.(Glu333Val) and c.1768 + 1 G > A, respectively. Second, GSD Ia is the most common type of hepatic GSD in Saudi Arabia (37%), similar to the data from Japan. Brazil, and China [6,15], in contrast to Iran and Egypt, where GSD III is the most predominant GSD [9,11], and GSD Ib in the Serbian population [16]. Third, 19 of the 24 GSD Ia cases (80%) originated from the southwestern region of Saudi Arabia, and the missense homozygous variant p.(Arg83Cys) was the predominant cause in 13 of the 19 cases (68%) from 12 unrelated families, suggesting that this variant came from a common ancestor, i.e., “founder in nature”. Furthermore, we identified clustering of GDS IV cases only in Al Madinah AlMunawarah Province due to a missense variant p.(Glu333Val) affecting 5 unrelated families from a single tribe that has not previously been reported in other populations, strongly suggesting that this variant is a founder in nature. Identification of the predominant gene variants and their geographic distribution in any population is likely to facilitate rapid molecular analysis by future targeting of that specific mutation. Another important finding from our data is that a single patient with GSD IXa, which was previously recognized to cause mild liver disease, developed cirrhosis as early as 3 years of age.

In addition to the abovementioned founder variants, our study cohort also revealed another two likely founder variants in *G6PC1* [c.59A > G, p.(Gln20Arg)] and *PYGL* [c.1768 + 1 G > A]. Recurrent founder mutations occur in populations with high consanguinity, such as Saudi Arabia, where the prevalence of consanguineous marriage is almost 60% [17], which presents a major risk factor for autosomal recessive diseases. Consanguinity was reported in 75% of our study cohort, and 28% reported a history of GSD in at least one close relative. The founder variants vary across different ethnic groups, where there is a high rate of consanguinity, such as the “4455delT” variant in the *AGL* gene (GSD III), which occurs with high frequency in the Tunisian population [10], and “W1327X” known as the disease-causing variant for GSD III in the Turkish population and German–Ukraine patients [18,19]. The most common founder variant in our study cohort [p.(Arg83Cys) affecting the *G6PC1* gene] is not unique to the Saudi population and was found in several populations [7,20–22], suggesting that this variant is of common ancestral origin worldwide. The p.Arg83Cys variant constituted 50% of the *G6PC* disease-causing variants in our study and in French and Tunisian patients [23,24], 80% of Sicilian, and 100% of *G6PC* variants in Ashkenazi Jewish patients [25,26].

The correlation between specific pathogenic variants and the clinical phenotype of GSD has been investigated in few studies. There is increasing evidence in the literature linking variants in *PHKG2* with more severe phenotypes than variants in the *PHKA2* and *PHKB* genes, which are usually associated with milder phenotypes [5]. Several studies have shown that GSD III lacks clear links between the genotype and clinical phenotype [27–29]; however, an association between exon 3 mutations and GSD IIIb (only the liver is involved) has been reported previously [30,31]. According to our data, the two founder variants in *G6PC* [p.(Arg83Cys) and p.(Gln20Arg), constituting 75% of all mutations in all 24 GSD Ia patients], were associated with a severe phenotype. The p.(Arg83Cys) variant is located in the active center of the enzyme G6 Pase and presented no detectable activity in transient expression assays [32]. The second variant [c.59A > G, p. (Gln20Arg)] is a nonhelical mutation that also presented no detectable G6 Pase activity [32]. The complete absence of enzyme activity as a result of both variants might explain the resulting severe phenotype in all our 18 patients harboring the 2 founder variants. On the other hand, the variant c.770C > T, p.(Pro257Leu) led to a mild phenotype in 2 siblings (hepatomegaly, no hypoglycemia, normal uric acid, nearly normal lactate and no kidney involvement) because this mutation retained residual phosphohydrolase activity (6.1% of the wild type) [33].

According to our data, the 2 missense variants in the *AGL* gene led to a mild–intermediate phenotype, and the nonmissense variants resulting from splicing, frameshift, or nonsense modifications resulting in a truncated protein led to a severe phenotype. We hypothesize that missense mutations in the *AGL* gene cause a minor reduction in glycogen debranching enzyme activity, whereas nonmissense variants lead to a marked reduction in enzyme activity. All the variants in the *G6PC1* gene led to a severe phenotype, except for the missense variant p.(Pro257Leu) in 2 siblings, which led to a mild phenotype over a 6- and 13-year follow-up period. There was no evidence of a genotype‒phenotype correlation within GSD I(a) patients when the two most common G6PC1 missense variants were compared (Supplementary S1 Table). However, long-term, natural history, large multicenter studies are needed to better understand the relationships between genotype and clinical presentation and outcome.

Compared with other types of hepatic GSD, GSD VI is usually mild with a benign course, and long-term complications, if they occur, are the exception. Consistent with the literature, all 7 unrelated cases of GSD VI in our study, which were from various regions in Saudi Arabia, manifested mild phenotypes, and none developed major complications over a median 10-year follow-up period. Only one patient developed mild asymptomatic cardiomyopathy, which has rarely been described [34]. However, in the literature, more severe cases with recurrent hypoglycemia, liver cirrhosis or developmental delay have been reported [35–37]. In a systematic literature review of 63 patients with GSD VI and 37 patients who underwent liver biopsy, fibrosis of various degrees was found in 32%, and early cirrhosis was diagnosed in approximately 10% of patients as early as preschool age [38]. Like in GSD VI, in the past, the clinical picture of GSD IXa was considered a benign and self-limited disorder; however, more recent reports linked GSD IXa with a greater prevalence of fibrotic or cirrhotic changes than did GSD VI [34,39,40]. Our single case of GSD IXa associated with early cirrhotic changes on liver biopsy at 3 years of age supports this observation; therefore, we highly recommend close monitoring for long-term liver complications in patients with GSD IXa. Unlike GSD IXa and GSD IXb, GSD IXc [due to deficiency of the γ subunit of phosphorylase kinase (PK) enzyme, encoded by the *PHKG2* gene) is a more severe disorder with a high prevalence of liver fibrosis that can progress to cirrhosis during childhood and the need for LT [5]. Furthermore, there is a report of hepatocellular carcinoma development in a 27-year-old man with GSD IXc [5]. On the other hand, no patients with GSD VI, GSD IXa or GSD IXb were reported to have developed liver failure or hepatocellular carcinoma and received a liver graft. Two of our 4 patients with GSD IXc developed advanced liver fibrosis during early childhood, as evidenced by the cirrhotic changes in the liver biopsy in one patient at 3 years of age and the development of portal hypertension in the other patient by 5 years of age. These findings provide further evidence that GSD IXc patients with mutations in the γ subunit of the PK enzyme suffer from more severe liver disease at an earlier age than patients with mutations in the α and β subunits encoded by the *PHKA2* and *PHKB* genes, respectively. A possible explanation for this disparity in severity between GSD IXc patients and GSD IXa and GSD IXb patients is that the γ subunit plays an important role in PK enzyme functionality because it houses the catalytic site, whereas the other subunits are involved in regulation [41].

Although our study provides valuable insights into the clinical and molecular characteristics of hepatic GSD in Saudi Arabia, it has several limitations. In addition to the retrospective design of the study, which has inherent limitations, the lack of consistency in assessment and monitoring for complications/comorbidities via standardized and systematic methods (e.g., regular performance of echocardiogram, neuromuscular/neurodevelopmental evaluation) precluded proper documentation of cardiac, neurodevelopmental, and muscular involvement. Histopathological examination of the liver was available for only 20% of the 65 patients, which might have led to an underestimation of the frequency of liver fibrosis reported in our study. Furthermore, the median follow-up time in our cohort was relatively short (≈ 9 years) and did not extend beyond 20 years of age. All these factors could have contributed to an underestimation of long-term complications. Another limitation is the limited availability of dietary management data, which has precluded assessment of the effects of dietary therapy on liver function tests and the occurrence of complications. Furthermore, the small sample size for some individual GSD subtypes did not allow for a meaningful comparative analysis to evaluate genotype‒phenotype correlations.

In conclusion, our results identified founder variants and provided geographic mapping of hepatic GSD in Saudi Arabia, which is likely to facilitate rapid molecular analysis by future targeting of that specific mutation. Longitudinal, prospective, multicenter studies are needed to better understand the natural history of hepatic GSD, perform regular surveillance for the development of hepatic and extrahepatic complications, evaluate “genotype‒phenotype correlations” among several variants leading to GSD, and examine the effects of various treatment approaches and proper metabolic management. The results from these studies will enable physicians to provide personalized care and appropriate family counseling.

## Supporting information

S1 Fig Hepatic adenoma in a 17-year-old girl with GSD Ib. (A) Tissue biopsy from the resected hepatic adenoma (arrow) is sharply demarcated from the adjacent nontumor liver tissue (arrowhead) (hematoxylin‒eosin, magnification 40x). (B) Tissue biopsy from a hepatic adenoma consisting of benign hepatocytes without portal tracts (hematoxylin‒eosin, magnification 40x). (C) Focal hypoechoic image of a 3 cm × 4 cm lesion on ultrasound of the liver. (D) A hyperdense rounded lesion on CT of the liver.
(JPG)

S2 Fig Liver biopsy from a patient with GSD Ib performed at the age of 8 years. (A) Glycogenic hepatocyte distension accompanied by the presence of glycogenated nuclei (arrow) and mild macrovesicular steatosis (arrowhead) (hematoxylin‒eosin, magnification 100x). (B) No significant fibrosis (arrow) (Masson’s trichrome stain, magnification 100x).
(JPG)

S3 Fig Liver biopsy from a patient with GSD VI performed at 8 years of age. (A) Hepatocytes appear enlarged and pale with cytoplasmic clearing (arrow) secondary to marked accumulation of glycogen (hematoxylin‒eosin, magnification 100x). (B) Stage 1 fibrosis (arrow) [Masson’s trichrome stain] and macroscopic steatosis (arrowhead).
(JPG)

S4 Fig Liver biopsy from a patient with GSD XIa performed at 3 years of age (A) Enlarged hepatocytes with clear cytoplasm (arrow). Some hepatocytes have light eosinophilic haziness to their cytoplasm (arrowhead). Both appearances can occur during hepatocyte overglycogenation (hematoxylin‒eosin, magnification 100x). (B) Bridging fibrosis (arrow), Stage 3/4 (Masson’s trichrome stain).
(JPG)

S5 Fig Distribution of the GSD subtypes and variants among children in Saudi Arabia. (TIF)

S1 Table Comparison of two G6PC1 gene variants. (DOCX)

S2 Table Histopathological features of 13 GSD patients. (DOCX)

S1 Data GDS Data Set (Plos One). (XLSM)

## References

[1] Hicks J, Wartchow E, Mierau G. Glycogen storage diseases: a brief review and update on clinical features, genetic abnormalities, pathologic features, and treatment. Ultrastruct Pathol. 2011;35(5):183–96. doi: 10.3109/01913123.2011.601404 21910565

[2] Kanungo S, Wells K, Tribett T, El-Gharbawy A. Glycogen metabolism and glycogen storage disorders. Ann Transl Med. 2018;6(24):474. doi: 10.21037/atm.2018.10.59 30740405 PMC6331362

[3] Szymańska E, Jóźwiak-Dzięcielewska DA, Gronek J, Niewczas M, Czarny W, Rokicki D, et al. Hepatic glycogen storage diseases: pathogenesis, clinical symptoms and therapeutic management. Arch Med Sci. 2019;17(2):304–13. doi: 10.5114/aoms.2019.83063 33747265 PMC7959092

[4] Grünert SC, Hannibal L, Spiekerkoetter U. The Phenotypic and Genetic Spectrum of Glycogen Storage Disease Type VI. Genes (Basel). 2021;12(8):1205. doi: 10.3390/genes12081205 34440378 PMC8391619

[5] Fernandes SA, Cooper GE, Gibson RA, Kishnani PS. Benign or not benign? Deep phenotyping of liver Glycogen Storage Disease IX. Mol Genet Metab. 2020;131(3):299–305. doi: 10.1016/j.ymgme.2020.10.004 33317799 PMC7953588

[6] Kido J, Nakamura K, Matsumoto S, Mitsubuchi H, Ohura T, Shigematsu Y, et al. Current status of hepatic glycogen storage disease in Japan: clinical manifestations, treatments and long-term outcomes. J Hum Genet. 2013;58(5):285–92. doi: 10.1038/jhg.2013.17 23486339

[7] Sperb-Ludwig F, Pinheiro FC, Bettio Soares M, Nalin T, Ribeiro EM, Steiner CE, et al. Glycogen storage diseases: Twenty-seven new variants in a cohort of 125 patients. Mol Genet Genomic Med. 2019;7(11):e877. doi: 10.1002/mgg3.877 31508908 PMC6825860

[8] Roscher A, Patel J, Hewson S, Nagy L, Feigenbaum A, Kronick J, et al. The natural history of glycogen storage disease types VI and IX: Long-term outcome from the largest metabolic center in Canada. Mol Genet Metab. 2014;113(3):171–6. doi: 10.1016/j.ymgme.2014.09.005 25266922

[9] Zahra B, Fatih E, Bita G, Mohammad Hadi I, Mahmood H, Seyed Mohsen D, et al. Clinical and genetic spectrum of glycogen storage disease in Iranian population using targeted gene sequencing. Sci Rep. 2021;11(1):7040. doi: 10.1038/s41598-021-94296-0 ; . 33782433 PMC8007705

[10] Mili A, Ben Charfeddine I, Mamaï O, Abdelhak S, Adala L, Amara A, et al. Molecular and biochemical characterization of Tunisian patients with glycogen storage disease type III. J Hum Genet. 2012;57(3):170–5. doi: 10.1038/jhg.2011.122 22089644

[11] El-Karaksy H, Anwar G, El-Raziky M, Mogahed E, Fateen E, Gouda A, et al. Glycogen storage disease type III in Egyptian children: a single centre clinico-laboratory study. Arab J Gastroenterol. 2014;15(2):63–7. doi: 10.1016/j.ajg.2014.01.013 25097048

[12] Albash B, Imtiaz F, Al-Zaidan H, Al-Manea H, Banemai M, Allam R, et al. Novel PHKG2 mutation causing GSD IX with prominent liver disease: report of three cases and review of literature. Eur J Pediatr. 2014;173(5):647–53. doi: 10.1007/s00431-013-2223-0 24326380

[13] Basit S, Malibari O, Al Balwi AM, Abdusamad F, Abu Ismail F. A founder splice site mutation underlies glycogen storage disease type 3 in consanguineous Saudi families. Ann Saudi Med. 2014;34(5):390–5. doi: 10.5144/0256-4947.2014.390 25827695 PMC6074555

[14] Richards S, Aziz N, Bale S, Bick D, Das S, Gastier-Foster J, et al. Standards and guidelines for the interpretation of sequence variants: a joint consensus recommendation of the American College of Medical Genetics and Genomics and the Association for Molecular Pathology. Genet Med. 2015;17(5):405–24. doi: 10.1038/gim.2015.30 25741868 PMC4544753

[15] Liang Y, Du C, Wei H, Zhang C, Zhang M, Hu M, et al. Genotypic and clinical analysis of 49 Chinese children with hepatic glycogen storage diseases. Mol Genet Genomic Med. 2020;8(10):e1444. doi: 10.1002/mgg3.1444 32772503 PMC7549605

[16] Skakic A, Djordjevic M, Sarajlija A, Klaassen K, Tosic N, Kecman B, et al. Genetic characterization of GSD I in Serbian population revealed unexpectedly high incidence of GSD Ib and 3 novel SLC37A4 variants. Clin Genet. 2018;93(2):350–5. doi: 10.1111/cge.13093 28685844

[17] el-Hazmi MA, al-Swailem AR, Warsy AS, al-Swailem AM, Sulaimani R, al-Meshari AA. Consanguinity among the Saudi Arabian population. J Med Genet. 1995;32(8):623–6. doi: 10.1136/jmg.32.8.623 7473654 PMC1051637

[18] Schoser B, Gläser D, Müller-Höcker J. Clinicopathological analysis of the homozygous p.W1327X AGL mutation in glycogen storage disease type 3. Am J Med Genet A. 2008;146A(22):2911–5. doi: 10.1002/ajmg.a.32529 18924225

[19] Aoyama Y, Ozer I, Demirkol M, Ebara T, Murase T, Podskarbi T, et al. Molecular features of 23 patients with glycogen storage disease type III in Turkey: a novel mutation p.R1147G associated with isolated glucosidase deficiency, along with 9 AGL mutations. J Hum Genet. 2009;54(11):681–6. doi: 10.1038/jhg.2009.100 19834502

[20] Chou JY, Jun HS, Mansfield BC. Glycogen storage disease type I and G6Pase-β deficiency: etiology and therapy. Nat Rev Endocrinol. 2010;6(12):676–88. doi: 10.1038/nrendo.2010.189 20975743 PMC4178929

[21] Matern D, Seydewitz HH, Bali D, Lang C, Chen Y-T. Glycogen storage disease type I: diagnosis and phenotype/genotype correlation. Eur J Pediatr. 2002;161(1):S10–9. doi: 10.1007/bf02679989 12373566

[22] Al-Shamsi A, Hertecant JL, Al-Hamad S, Souid A-K, Al-Jasmi F. Mutation Spectrum and Birth Prevalence of Inborn Errors of Metabolism among Emiratis: A study from Tawam Hospital Metabolic Center, United Arab Emirates. Sultan Qaboos Univ Med J. 2014;14(1):e42-9. doi: 10.12816/0003335 24516753 PMC3916276

[23] Barkaoui E, Cherif W, Tebib N, Charfeddine C, Ben Rhouma F, Azzouz H, et al. Mutation spectrum of glycogen storage disease type Ia in Tunisia: implication for molecular diagnosis. J Inherit Metab Dis. 2007;30(6):989. doi: 10.1007/s10545-007-0737-1 18008183

[24] Trioche P, Francoual J, Chalas J, Capel L, Lindenbaum A, Odièvre M, et al. Genetic heterogeneity of glycogen storage disease type Ia in France: a study of 48 patients. Hum Mutat. 2000;16(5):444. doi: 10.1002/1098-1004(200011)16:5<444::AID-HUMU10>3.0.CO;2-F 11058903

[25] Stroppiano M, Regis S, DiRocco M, Caroli F, Gandullia P, Gatti R. Mutations in the glucose-6-phosphatase gene of 53 Italian patients with glycogen storage disease type Ia. J Inherit Metab Dis. 1999;22(1):43–9. doi: 10.1023/a:1005495131118 10070617

[26] Ekstein J, Rubin BY, Anderson SL, Weinstein DA, Bach G, Abeliovich D, et al. Mutation frequencies for glycogen storage disease Ia in the Ashkenazi Jewish population. Am J Med Genet A. 2004;129A(2):162–4. doi: 10.1002/ajmg.a.30232 15316959

[27] Sentner CP, Hoogeveen IJ, Weinstein DA, Santer R, Murphy E, McKiernan PJ, et al. Glycogen storage disease type III: diagnosis, genotype, management, clinical course and outcome. J Inherit Metab Dis. 2016;39(5):697–704. doi: 10.1007/s10545-016-9932-2 27106217 PMC4987401

[28] Lucchiari S, Fogh I, Prelle A, Parini R, Bresolin N, Melis D, et al. Clinical and genetic variability of glycogen storage disease type IIIa: seven novel AGL gene mutations in the Mediterranean area. Am J Med Genet. 2002;109(3):183–90. doi: 10.1002/ajmg.10347 11977176

[29] Lucchiari S, Donati MA, Melis D, Filocamo M, Parini R, Bresolin N, et al. Mutational analysis of the AGL gene: five novel mutations in GSD III patients. Hum Mutat. 2003;22(4):337. doi: 10.1002/humu.9177 12955720

[30] Shen J, Bao Y, Liu HM, Lee P, Leonard JV, Chen YT. Mutations in exon 3 of the glycogen debranching enzyme gene are associated with glycogen storage disease type III that is differentially expressed in liver and muscle. J Clin Invest. 1996;98(2):352–7. doi: 10.1172/JCI118799 8755644 PMC507437

[31] Goldstein JL, Austin SL, Boyette K, Kanaly A, Veerapandiyan A, Rehder C, et al. Molecular analysis of the AGL gene: identification of 25 novel mutations and evidence of genetic heterogeneity in patients with Glycogen Storage Disease Type III. Genet Med. 2010;12(7):424–30. doi: 10.1097/GIM.0b013e3181d94eaa 20648714

[32] Lei KJ, Pan CJ, Shelly LL, Liu JL, Chou JY. Identification of mutations in the gene for glucose-6-phosphatase, the enzyme deficient in glycogen storage disease type 1a. J Clin Invest. 1994;93(5):1994–9. doi: 10.1172/JCI117192 8182131 PMC294308

[33] Takahashi K, Akanuma J, Matsubara Y, Fujii K, Kure S, Suzuki Y. Heterogeneous mutations in the glucose-6-phosphatase gene in Japanese patients with glycogen storage disease type Ia. Am J Med Genet. 2000;92(2):90–4. 10797430

[34] Roscher A, Patel J, Hewson S, Nagy L, Feigenbaum A, Kronick J, et al. The natural history of glycogen storage disease types VI and IX: Long-term outcome from the largest metabolic center in Canada. Mol Genet Metab. 2014;113(3):171–6. doi: 10.1016/j.ymgme.2014.09.005 25266922

[35] Beauchamp NJ, Taybert J, Champion MP, Layet V, Heinz-Erian P, Dalton A, et al. High frequency of missense mutations in glycogen storage disease type VI. J Inherit Metab Dis. 2007;30(5):722–34. doi: 10.1007/s10545-007-0499-9 17705025

[36] Zhan Q, Lv Z, Tang Q, Huang L, Chen X, Yang M, et al. Glycogen storage disease type VI with a novel PYGL mutation: Two case reports and literature review. Medicine (Baltimore). 2021;100(16):e25520. doi: 10.1097/MD.0000000000025520 33879691 PMC8078372

[37] Lu S-Q, Feng J-Y, Liu J, Xie X-B, Lu Y, Abuduxikuer K. Glycogen storage disease type VI can progress to cirrhosis: ten Chinese patients with GSD VI and a literature review. J Pediatr Endocrinol Metab. 2020;33(10):1321–33. doi: 10.1515/jpem-2020-0173 32892177

[38] Grünert SC, Hannibal L, Spiekerkoetter U. The Phenotypic and Genetic Spectrum of Glycogen Storage Disease Type VI. Genes (Basel). 2021;12(8):1205. doi: 10.3390/genes12081205 34440378 PMC8391619

[39] Johnson AO, Goldstein JL, Bali D. Glycogen storage disease type IX: novel PHKA2 missense mutation and cirrhosis. J Pediatr Gastroenterol Nutr. 2012;55(1):90–2. doi: 10.1097/MPG.0b013e31823276ea 21857251

[40] Tsilianidis LA, Fiske LM, Siegel S, Lumpkin C, Hoyt K, Wasserstein M, et al. Aggressive therapy improves cirrhosis in glycogen storage disease type IX. Mol Genet Metab. 2013;109(2):179–82. doi: 10.1016/j.ymgme.2013.03.009 23578772 PMC3672367

[41] Brushia RJ, Walsh DA. Phosphorylase kinase: the complexity of its regulation is reflected in the complexity of its structure. Front Biosci. 1999;4:D618-41. doi: 10.2741/brushia 10487978

