# Supplementary figures and images for: Clinical and molecular characterization of hepatic glycogen storage disease in Saudi Arabia
Source: PLoS One. 2025 Jul 31;20(7):e0329008. doi: 10.1371/journal.pone.0329008 (PMC12312935; doi:10.1371/journal.pone.0329008)

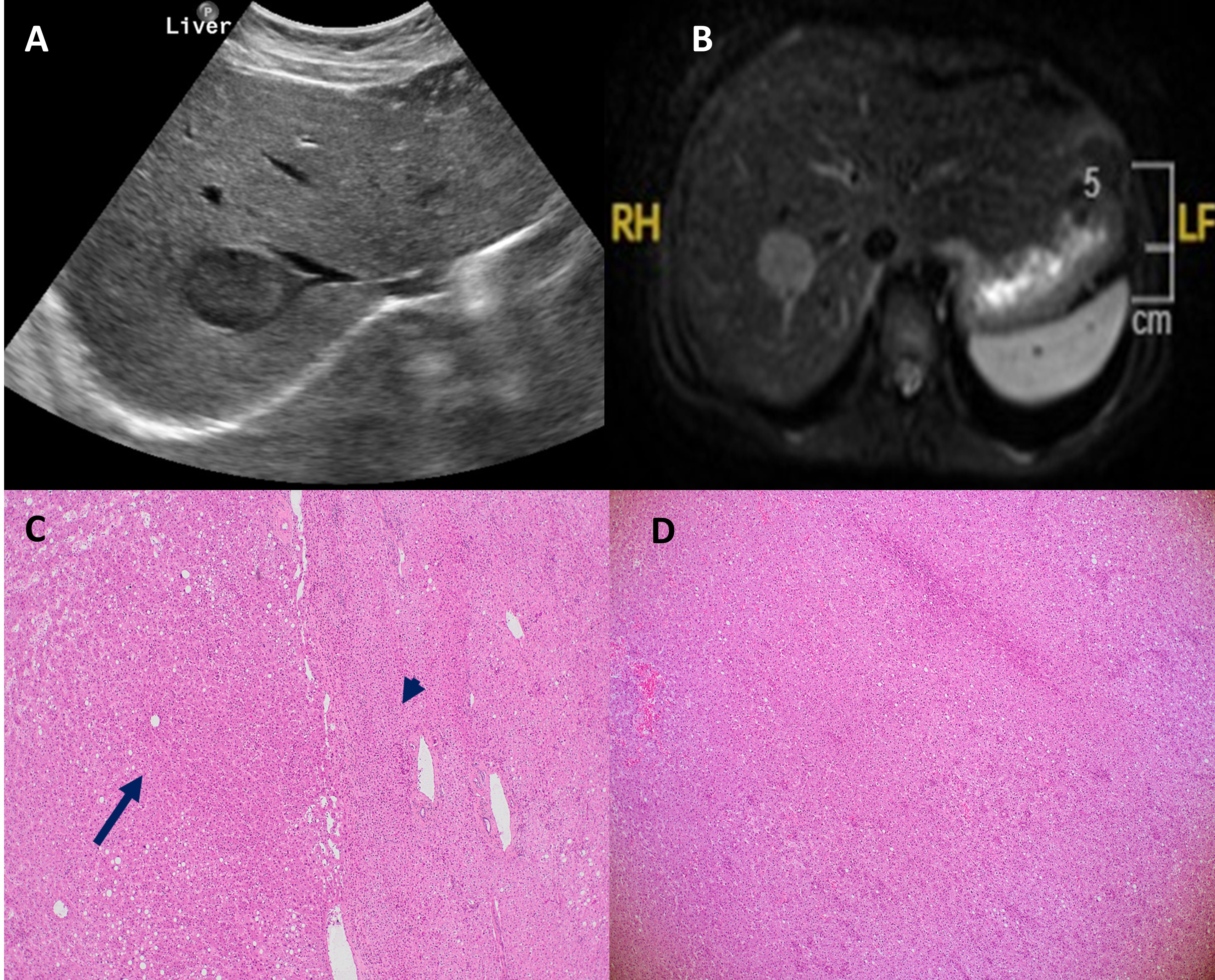

Supplement: S1 Fig — (A) Tissue biopsy from the resected hepatic adenoma (arrow) is sharply demarcated from the adjacent nontumor liver tissue (arrowhead) (hematoxylin‒eosin, magnification 40x). (B) Tissue biopsy from a hepatic adenoma consisting of benign hepatocytes without portal tracts (hematoxylin‒eosin, magnification 40x). (C) Focal hypoechoic image of a 3 cm × 4 cm lesion on ultrasound of the liver. (D) A hyperdense rounded lesion on CT of the liver. (JPG) [file pone.0329008.s001.jpg]

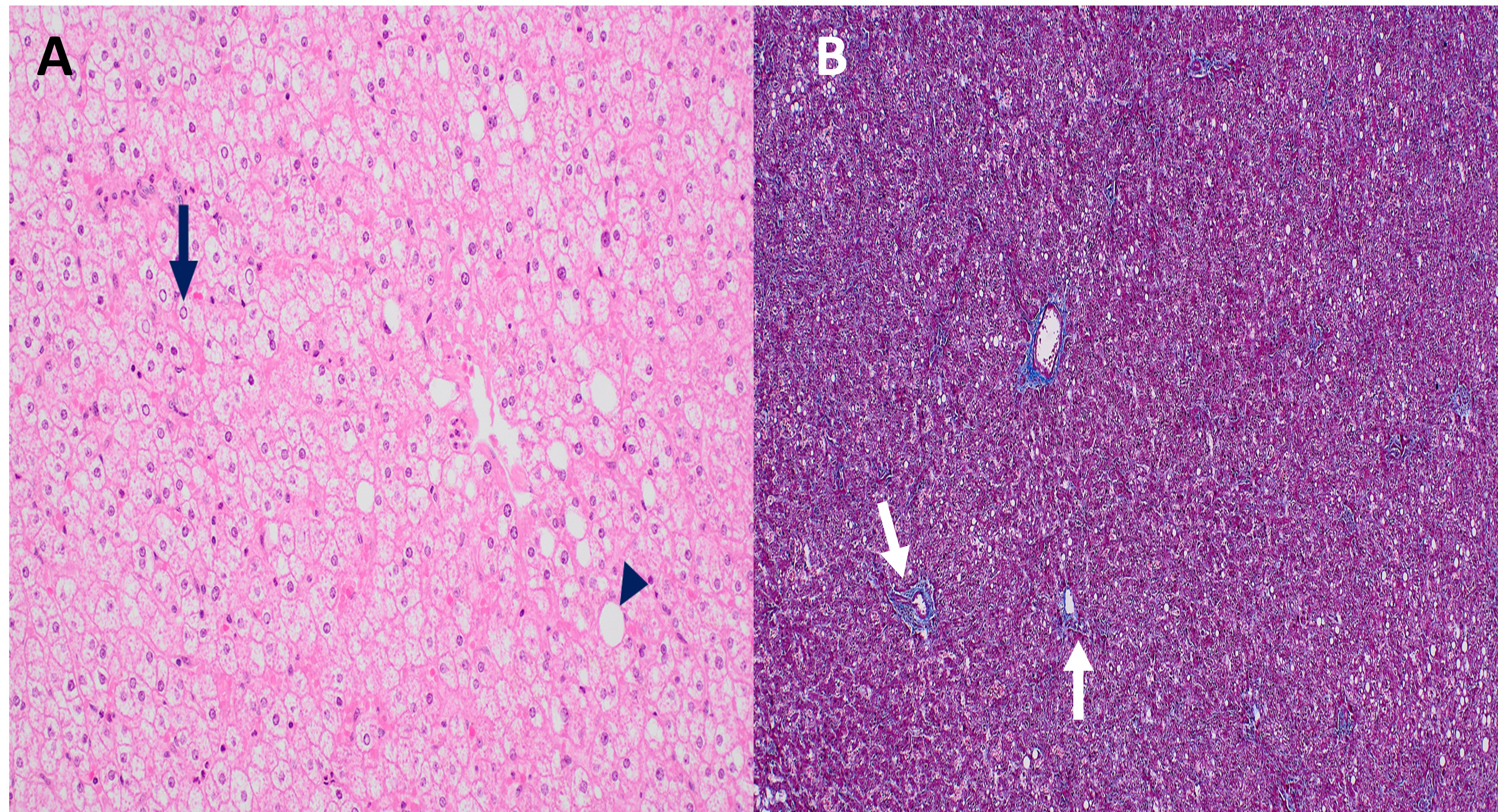

Supplement: S2 Fig — (A) Glycogenic hepatocyte distension accompanied by the presence of glycogenated nuclei (arrow) and mild macrovesicular steatosis (arrowhead) (hematoxylin‒eosin, magnification 100x). (B) No significant fibrosis (arrow) (Masson’s trichrome stain, magnification 100x). (JPG) [file pone.0329008.s002.jpg]

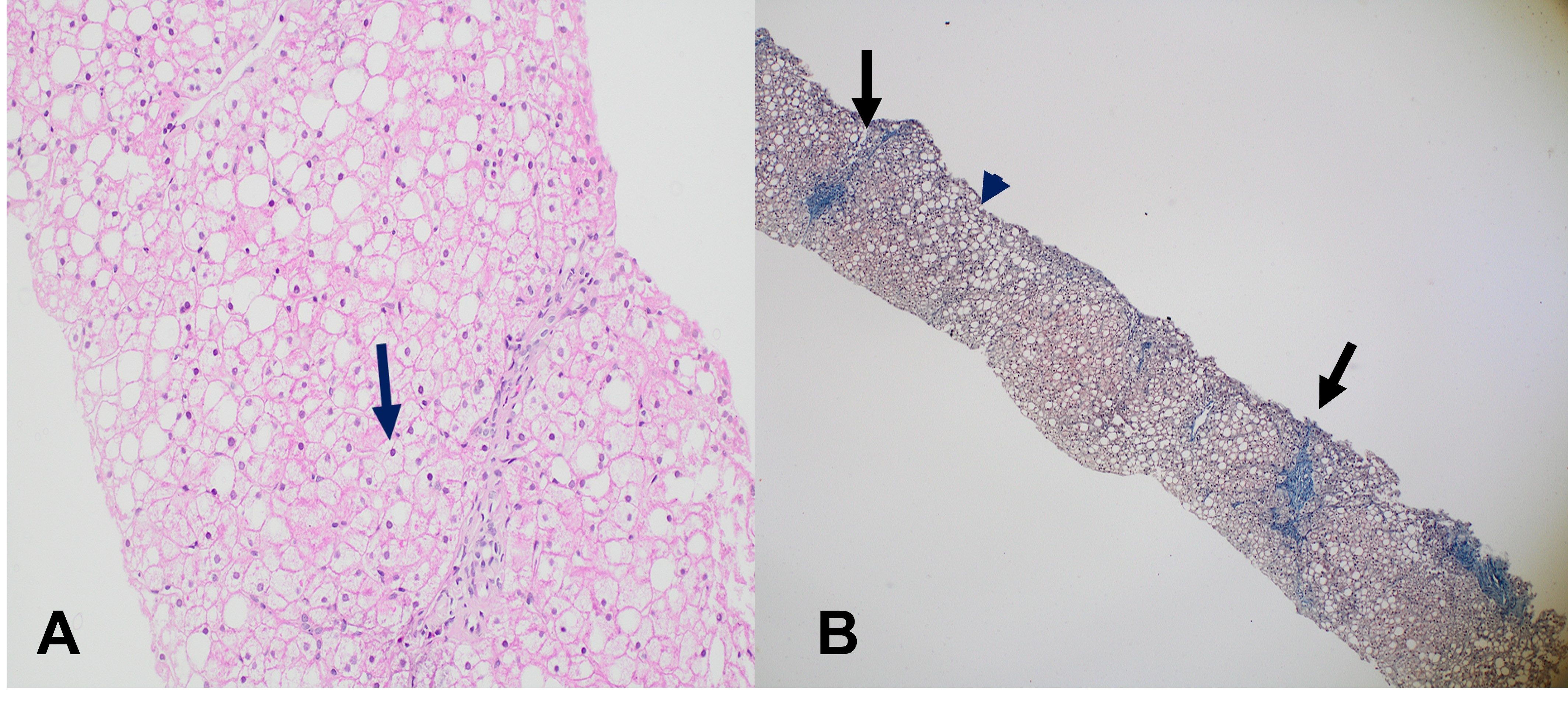

Supplement: S3 Fig — (A) Hepatocytes appear enlarged and pale with cytoplasmic clearing (arrow) secondary to marked accumulation of glycogen (hematoxylin‒eosin, magnification 100x). (B) Stage 1 fibrosis (arrow) [Masson’s trichrome stain] and macroscopic steatosis (arrowhead). (JPG) [file pone.0329008.s003.jpg]

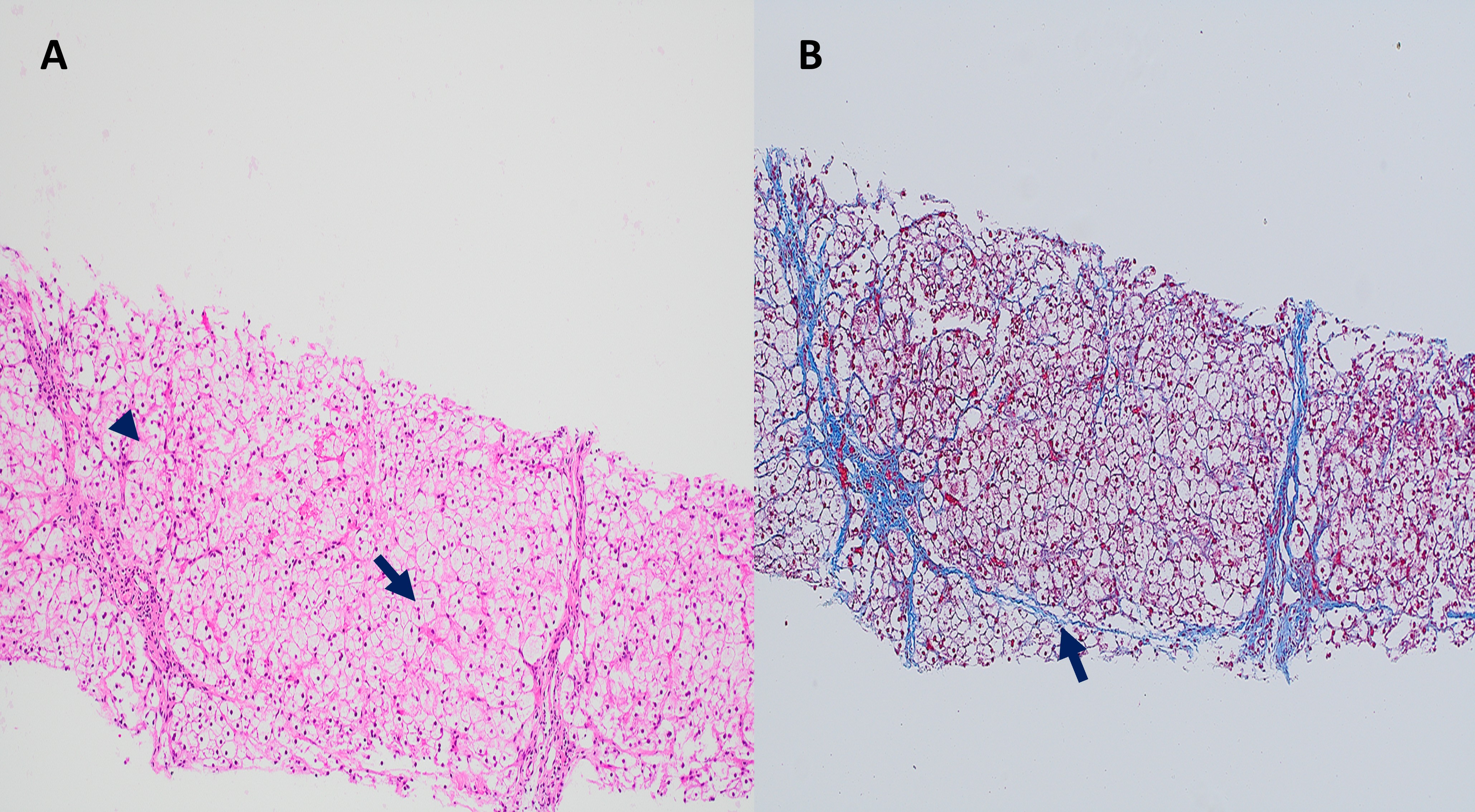

Supplement: S4 Fig — Some hepatocytes have light eosinophilic haziness to their cytoplasm (arrowhead). Both appearances can occur during hepatocyte overglycogenation (hematoxylin‒eosin, magnification 100x). (B) Bridging fibrosis (arrow), Stage 3/4 (Masson’s trichrome stain). (JPG) [file pone.0329008.s004.jpg]

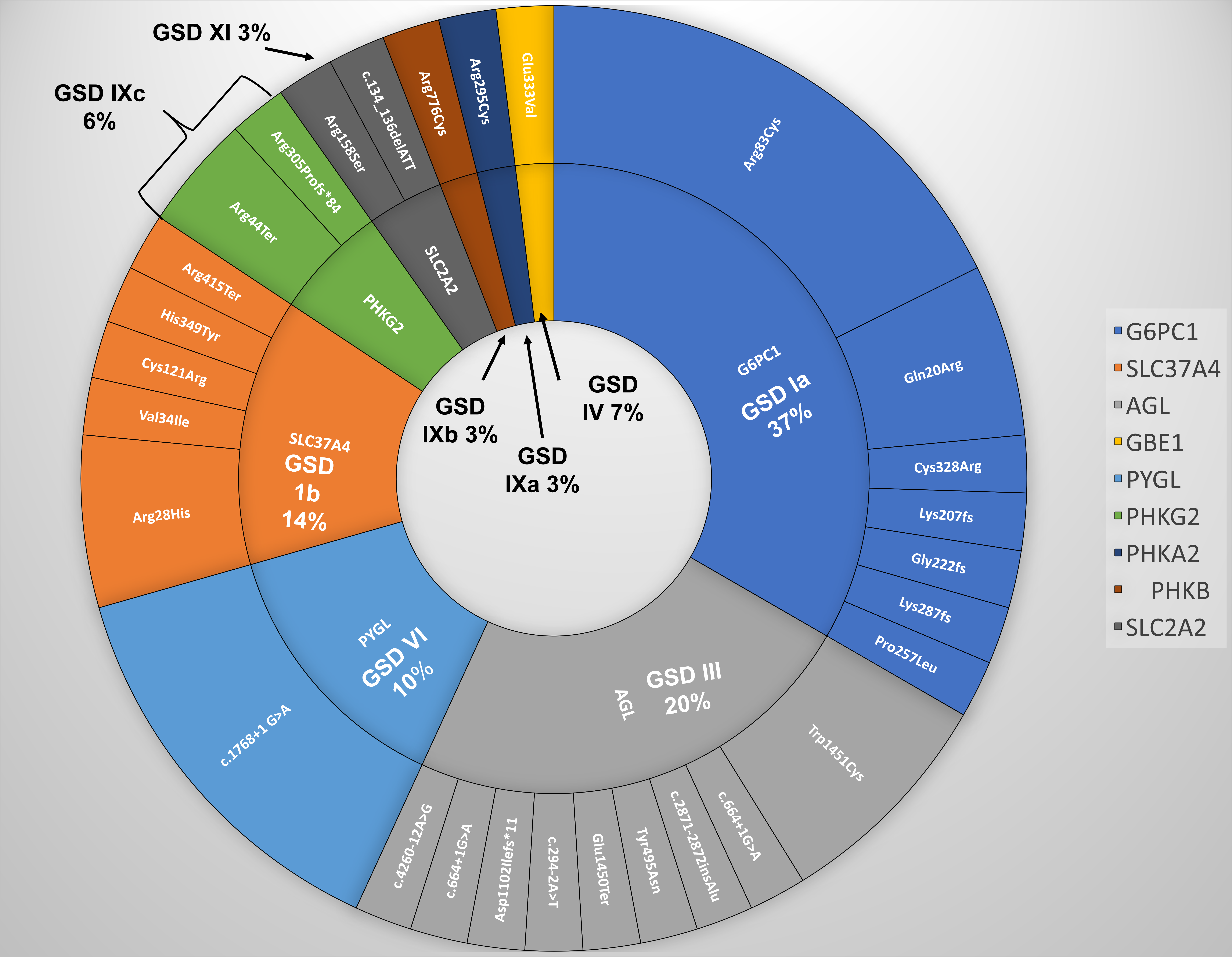

Supplement: S5 Fig — (TIF) [file pone.0329008.s005.tif]
