# Supplementary material for: Clinical and molecular characterization of hepatic glycogen storage disease in Saudi Arabia
Source: PLoS One. 2025 Jul 31;20(7):e0329008. doi: 10.1371/journal.pone.0329008 (PMC12312935; doi:10.1371/journal.pone.0329008)
Supplement: S1 Table — (DOCX) [file pone.0329008.s006.docx]

**Supplementary Table 1:** Comparison of the two most common *G6PC1 gene* variants

| Clinical Variable | c.247C>T,  p.(Arg83Cys)  N = 13 | c.59A>G, p.(Gln20Arg)  N = 5 |
| --- | --- | --- |
| Median Age at presentation (months) | 5 | 6.5 |
| Hepatomegaly% | 100% | 100% |
| Hypoglycemia% | 100% | 100% |
| Abdominal distention% | 100% | 100% |
| Enlarged echogenic kidney | 66.6% | 75% |
| ALT (I/U) < 55 u/l (median) | 102 | 89 |
| AST (I/U) < 34 (median) | 112 | 89 |
| Cholesterol (mmol/L)<4.4 (median) | 3.8 | 4 |
| Triglyceride (mmol/L) <1.7 mmol/L | 7.2 | 9 |
| Lactate (mmol/L) < 2.2 mmol/l (median) | 6 | 9 |
| Uric acid (µmol/L) 210-420 (median) | 433 | 338 |
| Liver transplant % | 2 (15%) | 1 (20%) |
