# Supplementary material for: Clinical and molecular characterization of hepatic glycogen storage disease in Saudi Arabia
Source: PLoS One. 2025 Jul 31;20(7):e0329008. doi: 10.1371/journal.pone.0329008 (PMC12312935; doi:10.1371/journal.pone.0329008)
Supplement: S2 Table — (DOCX) [file pone.0329008.s007.docx]

**Supplementary Table 2.** Histopathological features of 13 GSD patients

|  | **Type of GSD** | **Age at biopsy** | **Stage of Portal fibrosis** | **Distension of hepatocytes with glycogen** | **Glycogenated nuclei** | **Steatosis** |
| --- | --- | --- | --- | --- | --- | --- |
| **1** | GSD 1 (a) | na | 0 | Yes | Yes | Yes |
| **2** | GSD 1 (a) | 1 year | 0 | Yes | Yes | Yes |
| **3** | GSD 1 (a) | 10 months | 0 | Yes | Yes | Yes |
| **4** | GSD 1 (a) | 2.5 years | 1 | Yes | Yes | Yes |
| **5** | GSD 1 (b) | 8 years | 0 | Yes | Yes | Yes |
| **6** | GSD III | na | 3 | Yes | Yes | No |
| **7** | GSD III | 6 years | 4 | Yes | No | No |
| **8** | GSD VI | 2 years | 1 | Yes | No | Yes |
| **9** | GSD VI | 8 years | 1 | Yes | Yes | Yes |
| **10** | GSD VI | 3 years | 1 | Yes | No | No |
| **11** | GSD IX (*PHKG2*) | 1 year | 3-4 | Yes | No | No |
| **12** | GSD IX  (*PHKA2*) | 3 years | 3-4 | Yes | No | No |
| **13** | GSD IX  (*PHKB*) | 1.5 years | 2 | Yes | Yes | No |
